# Quantification, Sources, and Control of Ammonia Emissions in the Czech Republic

**DOI:** 10.1100/tsw.2001.382

**Published:** 2001-12-01

**Authors:** Marie Skybova

**Affiliations:** Ekotoxa Opava, Ltd, Czech Republic

**Keywords:** ammonia emissions, emission factors, breeding of cattle, pigs/swine, poultry, reducing emissions from agriculture, Europe

## Abstract

The exact quantification of ammonia (NH_3_) emissions is the basic presumption for the fulfilment of obligations set by the CLRTAP (Convention on Long Range Transboundary Air Pollution) Protocol which was signed by the Czech Republic in 1999. Most NH_3_ emissions in the Czech Republic are produced during the breeding of cattle, pigs, and poultry; therefore, determinating emission factors for these kinds of animals by studying their total number, type of breeding, and subsequent disposal of manure is the solution to the problem of NH_3_ emissions quantification. This paper summarizes the results of 4 years of research in this area, determining the emission factors and ways of decreasing emissions from the breeding of cattle, pigs, and poultry.

### INTRODUCTION

The Czech Republic signed the Protocol to Abate Acidification, Eutrophication, and Ground-Level Ozone in 1999. This protocol is one of the protocols of the Convention on Long Range Transboundary Air Pollution (CLRTAP). The protocol sets emission limits which should be reached by the year 2010 for four pollutants: sulphur, nitrogen oxides ($NO_x$), volatile organic compounds (VOCs), and ammonia ($NH_3$). The aim of this protocol is to limit and decrease emissions of those compounds which are the result of anthropogenic activities and have negative impacts on the health of people, natural ecosystems, materials, and crops. The following long-term objectives are the result of signing the protocol:

- To limit and decrease emissions of $NH_3$ resulting from anthropogenic activities while taking into account the achieved level of scientific research and development; and
- To not exceed emission limits for $NH_3$ set for the year 2010, which is 101 kt $NH_3$/year.

This emission limit represents a decrease of 35% compared to the situation in 1990 (Fig. 1).

The basic presumption for conducting this study was to identify the most accurate quantification of $NH_3$ emissions. Most of the emitted $NH_3$a in the Czech Republic is produced in the breeding of cattle, pigs, and poultry. The solution to the problem is to determinate the emission factors for those kinds of animals whose breeding is the main source of $NH_3$ in regard to their total number, type of breeding, and subsequent disposal of manure.

Ekotoxa Opava, Ltd. has dealt with this topic since 1997[1,2,3]. We have participated in Research and Development projects supported by the Czech Department of Environment. Currently we are participating in the project “Evaluation of the Readiness of the Czech Republic to Fulfil Requirements of the LRTAP Convention and EU Directions in Air Quality”. The aim of this project is to prepare a territorially structured balance of $NH_3$ emissions.

### EMISSION FACTORS

The emission factor for various kinds of domestic animals consists of several partial emission factors, which must be determined separately because each of them has its own specific

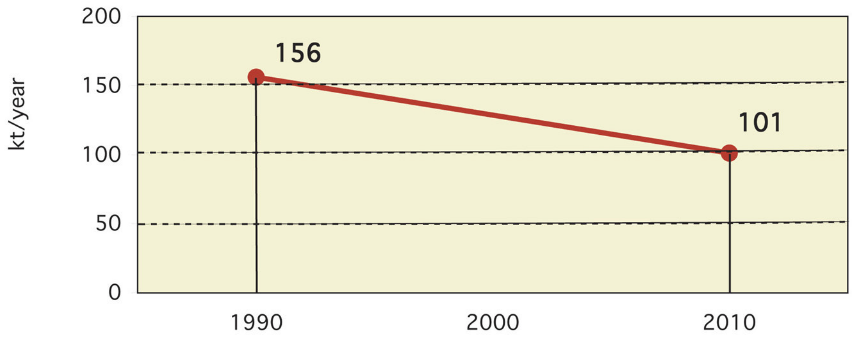

**FIGURE 1.** Required $NH_3$ emissions decrease in 20 years.

problems and variables of measurement. The types of emission factors are as follows:

- Emission factor from stables
- Emission factor from dung-yards, dung-pits, and transport of manure
- Emission factor from manure application
- Emission factor from the pasture period

The breeding of domestic animals is a system which is complicated to analyze. In this respect, it is necessary to prove the reliability of results on a statistically significant number of experiments. In much of the previous work, the emissions from stables and dung-yards/pits were regarded as relatively quantified. However, we find it neccessary to deal more with the emissions from manure application, emissions from the pasture period of breeding cattle, and those from breeding poultry. The emission factor from manure application depends on various conditions of the experiment, e.g., meteorological parameters, the time between the application and plowdown, the speed of plowdown, the age of manure, the amount of nitrogen components in manure, features of the soil where manure is applied, the system of monitoring, homogenization of samples, etc. Therefore, a significant number of experiments is needed to achieve accurate results.

### Determining Emission Factors from Stables

#### Method of Measurement

The emissions from stabling cattle and pigs were monitored by the mass balance method. Calculations were made using data of concentrations and the air flow in stables through ventilators, doors, and windows (Fig. 2).

#### Results of the Experiment

Experiments were carried out to set emission factors depending on age of animals and various conditions of stabling. There is an evident interdependence between the emission factor and the age and weight of animals. The emission factor increases with the age and weight of animals (Fig. 3, 4, and 5).

### Determining Emission Factors from Manure Application

#### Method of Measurement

The experiments were carried out using passive samplers with sulphuric acid. This part was inspired by the work of Vandre and

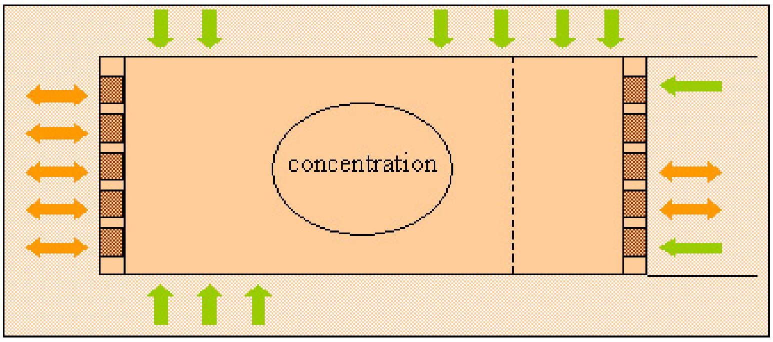

**FIGURE 2.** Scheme of emissions measurements from stables.

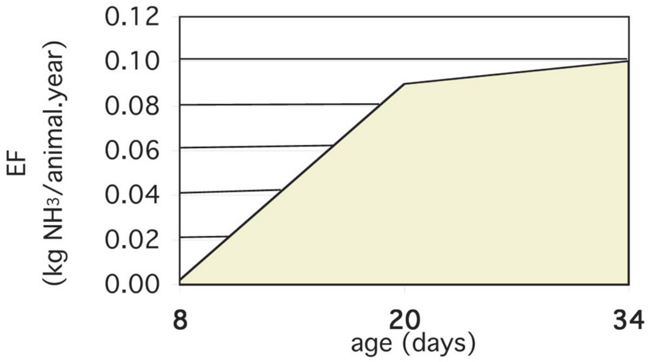

**FIGURE 3.** $NH_3$ emissions factors for broilers.

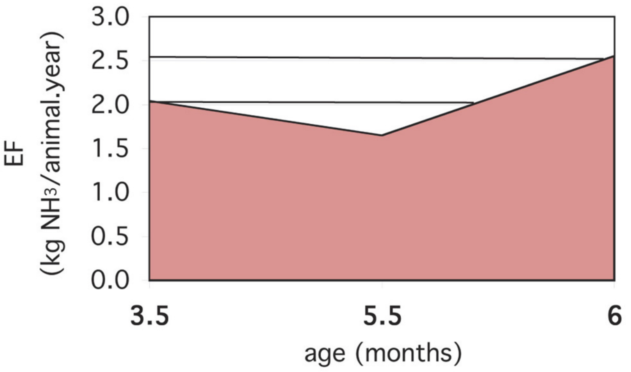

**FIGURE 4.** $NH_3$ emissions factors for swinery.

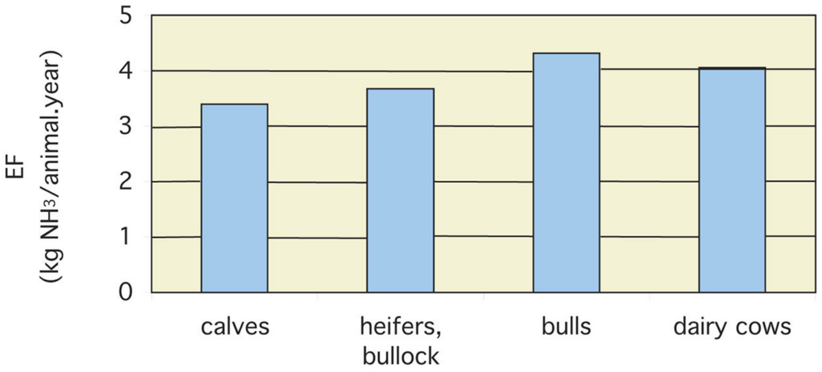

**FIGURE 5.** $NH_3$ emissions factors for cattle in stables.

Kaupenjohann[4] which was presented at the Nitrogen Conference in Great Britain in 1995. This report describes measuring of $NH_3$ emissions from manure application. The advantage of this method is its independence from meteorological conditions.

The indirect method is based on the comparison between unknown $NH_3$ release rates of manured plots (*Rm*) and $NH_3$ sources of known strength. In addition to experimental plots, standard plots are established and release $NH_3$ at known rates (*Rs*) during the experiment. Standard plots are provided with $NH_3$ gas cylinder, regulator, and flow counter. $NH_3$ is passed into a plastic tubing (commercial sewage tubing with plug connections; inside diameter 50 mm) near the soil surface. Air is pumped into the tubing as carrier gas. The gas mixture is released through small holes drilled into the tubing (diameter 0.5 mm), approaching a two-dimensional source by a network of point sources (4 sources per square meter) (Fig. 6).

On each plot, a $NH_3$ sensor is placed leeward near the ground (e.g., 0.25 m above ground). We used passive samplers collecting $NH_3$ in sulphuric acid (10 ml, 0.01 m). Samplers consisted of roofed polyethylene containers covered with gauze to keep insects away. $NH_3$ collected as ammonium ($NH_4^+$) is measured colorimetrically. These simple sensors provide a relative response to $NH_3$ concentration in the air. This is sufficient, as will be shown below. Background $NH_3$ concentration is measured windward. In case of calm or unsteady wind, sensors are placed in the middle of the plots, with background signals being measured at some distance from the plots.

The sensor signal of a standard plot (*Ss*) as well as that of a manured plot (*Sm*) may be related to the respective release rates (*Rs, Rm*) as follows:

$$Ss = Bs + psRs \qquad (1)$$

and

$$Sm = Bm + pmRm \qquad (2)$$

with *Bm, Bs* being the background signals of standard and manured plots, respectively, and *pm, ps* being the proportionality factors for $NH_3$ release from standard and manured plots, respectively.

$$pm = ps[4] \qquad (3)$$

and combine Eq. 1 and 2 to estimate *Rm* from *Rs* and background corrected sensor signals:

$$Rm = Rs\frac{Sm - Bm}{Ss - Bs} \qquad (4)$$

### Results of the Experiment

In 1999 and 2000, experiments relating to cattle manure and liquid manure from cattle and pigs were carried out. Emission factors from poultry were measured in 2001. Various methods of organic fertilizers plowdown were evaluated and compared. The interdependence between the application and plowdown of manure was discovered. This knowledge is important for minimizing $NH_3$ emissions and for optimizing good agricultural practice.

Comparisons were made to see if the direct injection of liquid manure is more advantageous than spraying and subsequent plowdown. After spraying the pig liquid manure on the field, the loss of ammoniacal nitrogen was 37% in 1 day, and nearly 100% in 1 week. The average time between spraying and plowdown is 3 h. During this time, about 16% of $NH_3$ escapes when manure is sprayed, while in cases of direct injection, the loss is only about 2% (see Fig. 7).

The biggest loss of $NH_3$ takes place in the first 4 h after the application of manure (see Fig. 8).

The application of solid manure with prompt plowdown is the most ecological as well as economical possibility. Therefore, cattle breeding without bedding is not a recommended practice (see Table 1 and Fig. 9).

There is no significant difference between the emissions from pigs/swine and cattle liquid manure. The type of manure (solid/liquid) is a more dominant difference influencing the emmissions than the species of animals producing this manure.

The variables of temperature when the manure was applied were also monitored (Fig. 10).

### Determining Emission Factors from Dung-Yards, Dung-Pits, and Transport of Manure

Monitoring of emissions from dung-yards and dung-pits was carried out afterwards. It involved manure and liquid manure. The published emission factors usually quantify emissions from

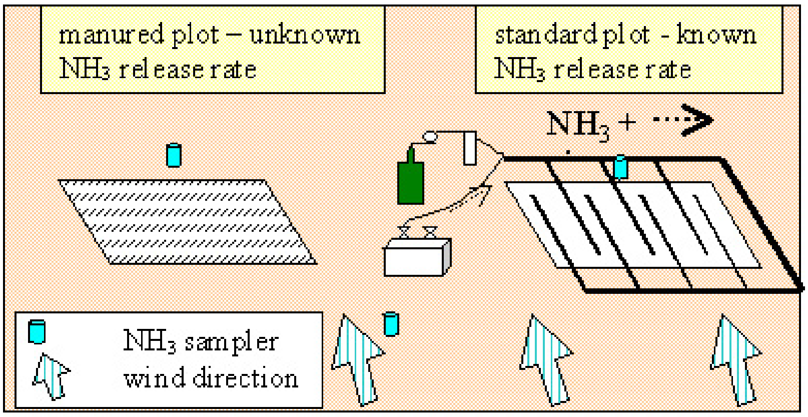

**FIGURE 6.** Scheme of equipment for indirect measurement of $NH_3$ emissions.

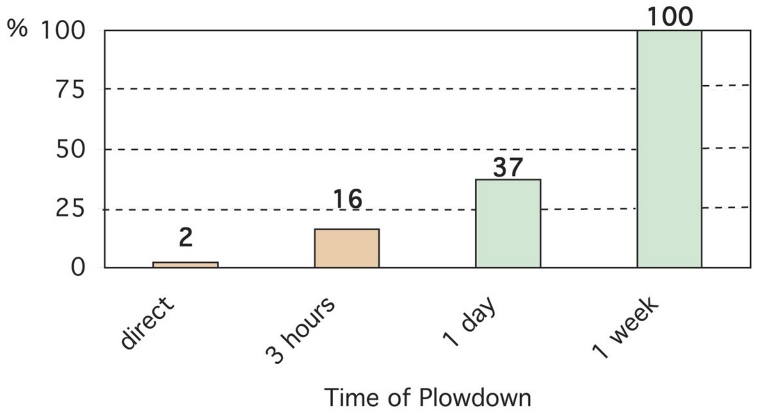

**FIGURE 7.** The interdependence between the escape of $NH_3$ and the time of plowdown – liquid manure.

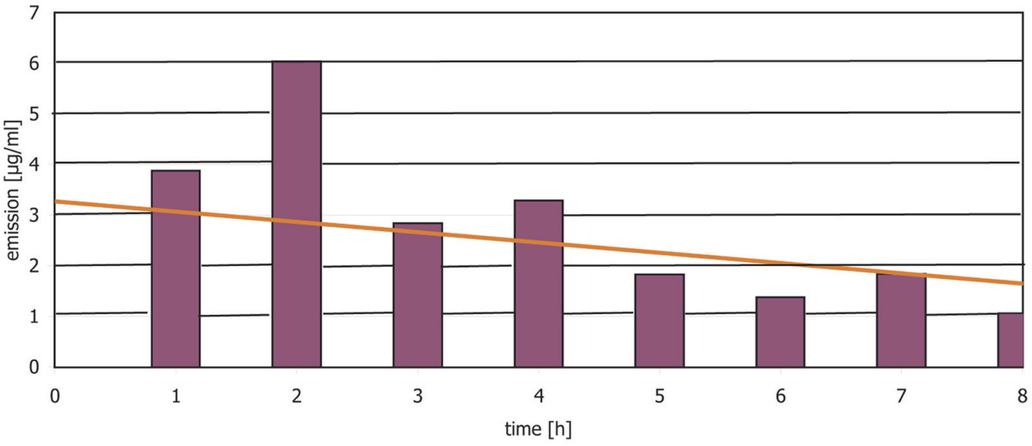

**FIGURE 8.** The interdependence between $NH_3$ emissions per hour and the time of plowdown – solid manure.

**TABLE 1**
**The Comparison of Emission Factors from the Application of Liquid and Solid Manures**

| Type of Manure | EF (kg $NH_3$/LU/year) | Time of Plowdown |
|---|---|---|
| Liquid manure | 10.4 | Injection |
| Solid manure | 3.51 | After 1 h |
| | 8.96 | After 2 h |
| | 11.56 | After 3 h |
| | 20.1 | After 8 h |

stables, dung-pits, manure application, and eventually the pasture period. But there is an additional manure handling, especially in cattle breeding. The calculation of this additional handling is a very complicated problem due to manipulation differences from farm to farm. It depends on the method of transloading and the number of transloadings between the stable and the place of manure application, and also on the size and area of the dung-yard/pit which affects the surface area from which $NH_3$ is emitted. Therefore, we have extended the program of emissions quantification with monitoring of the manure handling process, starting with the place of creation and finishing with the manure application on the field. The quantification was difficult,

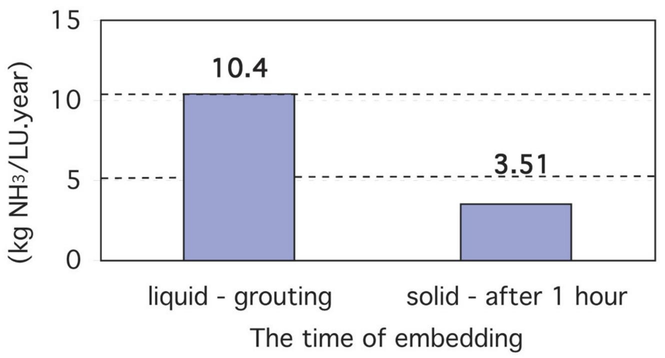

**FIGURE 9.** The comparison of emission factors from the application of liquid and solid manures.

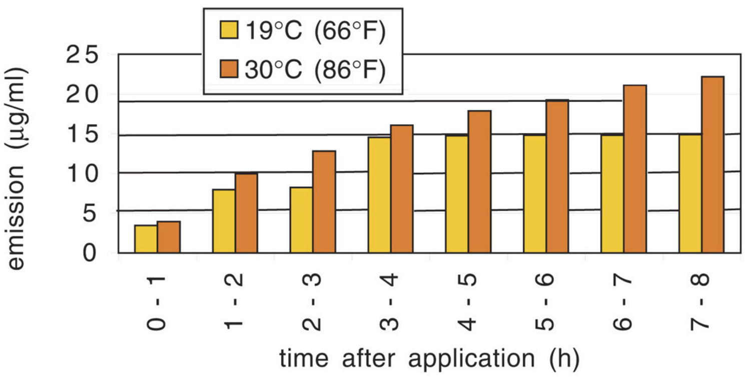

**FIGURE 10.** The interdependence between $NH_3$ emissions from manure application and outdoor temperature.

and in the end we also used the method of passive samplers. It is evident from the results that the emissions from transloading manure to a dung-yard, and during the initial hours after this transloading, are much higher than the emissions from clearing manure from the stable. The $NH_3$ concentrations during the clearing increase only very slightly above the level of emissions from stabling (Fig. 11).

### Determining Emission Factors from Pasture Period

The experimental quantification of the emission factor from the pasture period of cattle is not ready at present because there are many difficulties in creating an experimental design. We tried two methods: the first one was again the method of passive samplers which was successfully used in previous experiments. But it is evident from the results obtained from these experiments that the emission factor from cattle grazing is too low, especially in comparison with the results of balance method. It is necessary to continue experimenting and recording the amount of emissions, because at present more and more cattle are breeding outdoors in the Czech Republic.

We also tried to use monitoring chambers from the VUZT in Prague[5]. This method is based on the theoretical background of Svenson and Ferm[6]. It has been developed and tested since 1996, and it gives good results in monitoring emissions from

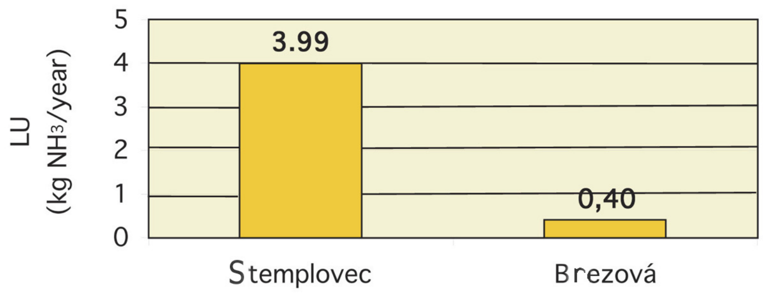

**FIGURE 11.** Specific emissions from a fresh dung-yard (Stemplovec) and an old dung-yard (Brezova).

manure applications and dung-yards. But the $NH_3$ concentrations during the pasture period are below the detection limit of the sensors.

### Experimentally Developed $NH_3$ Emissions Factors

The final set of experimentally developed emission factors is presented in Table 2 and Table 3. The emission factor from pasture period was calculated theoretically from a mass balance.

## TECHNOLOGICAL POSSIBILITIES OF DECREASING $NH_3$ EMISSIONS

The possibilities of decreasing $NH_3$ emissions from dung-yards/pits and manure applications are evident from the experiments that were carried out.

### Solid Manures

Deep bedding accumulates wastes and $NH_3$ emissions go on for longer than in the stable. Moreover, during a single transport and storage on a landfill (by the time of application), there is not a high loss of $NH_3$. Daily transport of waste to an open landfill with regular manipulation with manure shows higher $NH_3$ emissions due to a larger surface area exposed to the air. With regard to technological cycles in agriculture, there is no possibility of applying manure during the winter. With regard to the technology of manure spreading, the most important factor is the time of plowdown which must not be longer than 4 h after spreading.

### Liquid Manures

It is possible to decrease the loss of $NH_3$ by closing the storage pits as well as reducing the movement of liquid manure. It is important to achieve the least possible air circulation on the surface of liquid manure. This simple system is able to decrease $NH_3$ emissions by 70 to 90%, depending on the type of closing of storage pits. The lower pH, the smaller amount of $NH_3$ is present in a volatile nonionized form. It means that maintaining pH lower than 7 helps to decrease $NH_3$ emissions. It is possible to apply liquid manure under the surface of the soil by injection machines or by an immediate plowdown. These methods considerably decrease $NH_3$ emissions. In addition, there is another advantage of these methods, and that is the possibility to use them at the time of vegetation resulting in shorter periods during which the liquid manure has to be stored.

The experiment led to the conclusion that the application of solid manure with prompt plowdown is the most ecological as well as economical possibility. In this case, $NH_3$ emissions are lower than during the best application of liquid manure. In both cases, the application is better at lower temperatures.

## CONCLUSIONS

There has been an evident decrease of $NH_3$ emissions during the last 10 years in the Czech Republic (Fig. 12).

**TABLE 2**
**Experimentally Developed $NH_3$ Emissions Factors for Cattle and Pigs**

| | EF (kg $NH_3$/animal/year) | |
|---|---|---|
| **Category of Animals** | **Without Pasture Period** | **With Pasture Period** |
| Dairy cows | 22.6 | 25.8 |
| Calves, heifers, bulls | 11.0 | 10.9 |
| Pigs/swine (average) | 10.1 | — |
| Pigs/swine (50 to 120 kg) | 10.5 | — |
| Porkers (<50 kg) | 3.2 | — |

**TABLE 3**
**Experimentally Developed $NH_3$ Emissions Factors for Poultry**

| Category of Animals | EF (kg $NH_3$/animal/year) |
|---|---|
| Broilers on bedding | 0.10 |
| Turkeys on bedding | 0.46 |
| Hens and cocks on bedding — eggs for hatch | 0.47 |
| Layers in combined stable — cages, bedding | 0.36 |
| Layers in cages | 0.15 |

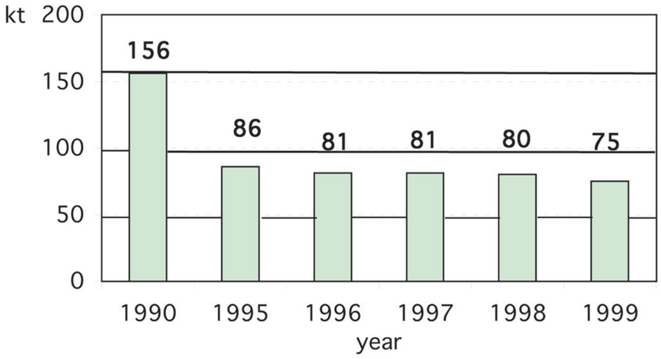

**FIGURE 12.** $NH_3$ emissions, Czech Republic.

The main reason is the continual decrease of number of domestic animals, installation of low emission technologies, and keeping good agricultural technologies during storage and application of manure. The large reduction of fertilizers consumption, including carbamide and DAM liquid fertilizer, has also had a considerable influence on the decrease of $NH_3$ emissions. This trend of decreasing emissions is positive, but in connection with emissions decreasing strategy, the main attention should be paid to the implementation of modern technologies. The system of monitoring and evaluating $NH_3$ emissions from agriculture has not been sufficiently integrated in the Czech legislation. Regular monitoring and sanctions for not keeping the defined indicators have not been solved either. Nowadays, good agricultural practice is influenced by the economical behavior of farmers (their aim is to keep the maximum amount of nitrogen in manure) rather than by the force of the Czech legislation. The emission taxes are based on the species and number of raised animals. This number is multiplied by the official emission factor per animal and year. Therefore, it is necessary to issue and distribute the advisory codex of good agricultural practice. When a balanced level of agricultural production is reached, the reduction of emmissions will be possible only by introducing technlogies with minimum $NH_3$ emissions both in vegetal and in animal productions.

| | | |
|---|---|---|
| Received: | July | 27, 2001 |
| Revised: | November | 9, 2001 |
| Accepted: | November | 10, 2001 |
| Published: | December | 1, 2001 |

### BIOSKETCH

**Marie Skybova** is Head of Environmental Monitoring Department at Ekotoxa Opava, s.r.o. – The Centre of Environment and Land Assessment. She holds a Dipl. Ing. Degree and is a graduate of The Institute of Chemical Technology, Prague. Her research interests include air pollution monitoring for towns, forests, and agricultural ecosystems; monitoring of $SO_2$, $NO_x$, $NH_3$, TSP, ozone, VOCs, and PAHs in emissions and wet atmospheric deposition; emissions of ammonia from agricultural sources; determination and verification of emission factors of $NH_3$ from breeding of cattle, pigs, and poultry; emissions from mobile sources and impact of transport on environment; monitoring of toxic materials in food chains; and risk and impact assesment of environment, calculation of damage in agriculture and forestry, and health risk of population.
